# Phase Gradients and Anisotropy of the Suprachiasmatic Network: Discovery of Phaseoids

**DOI:** 10.1523/ENEURO.0078-21.2021

**Published:** 2021-09-08

**Authors:** Tomoko Yoshikawa, Scott Pauls, Nicholas Foley, Alana Taub, Joseph LeSauter, Duncan Foley, Ken-Ichi Honma, Sato Honma, Rae Silver

**Affiliations:** 1 Organization for International Education and Exchange, University of Toyama, Toyama 930-8555, Japan; 2 Department of Mathematics, Dartmouth College, Hanover, NH 03755; 3 Department of Neuroscience and Zuckerman Mind Brain Behavior Institute, Columbia University, New York City, NY 10027; 4 Department of Neuroscience, Barnard College, New York City, NY 10027; 5 Department of Economics, New School for Social Research, New York City, NY 10011; 6 Research and Education Center for Brain Science, Hokkaido University, Sapporo 060-8638, Japan; 7 Center for Sleep and Circadian Rhythm Disorders, Sapporo Hanazono Hospital, Sapporo 064-0915, Japan; 8 Department of Psychology, Columbia University, New York City, NY 10027; 9 Department of Pathology and Cell Biology, Graduate teaching faculty, Columbia University Medical School, New York City, NY 10032

**Keywords:** circadian, phase waves, network, vasopressin, vasoactive intestinal peptide, oscillation

## Abstract

Biological neural networks operate at several levels of granularity, from the individual neuron to local neural circuits to networks of thousands of cells. The daily oscillation of the brain’s master clock in the suprachiasmatic nucleus (SCN) rests on a yet to be identified network of connectivity among its ∼20,000 neurons. The SCN provides an accessible model to explore neural organization at several levels of organization. To relate cellular to local and global network behaviors, we explore network topology by examining SCN slices in three orientations using immunochemistry, light and confocal microscopy, real-time imaging, and mathematical modeling. Importantly, the results reveal small local groupings of neurons that form intermediate structures, here termed “phaseoids,” which can be identified through stable local phase differences of varying magnitude among neighboring cells. These local differences in phase are distinct from the global phase relationship, namely that between individual cells and the mean oscillation of the overall SCN. The magnitude of the phaseoids’ local phase differences is associated with a global phase gradient observed in the SCN’s rostral-caudal extent. Modeling results show that a gradient in connectivity strength can explain the observed gradient of phaseoid strength, an extremely parsimonious explanation for the heterogeneous oscillatory structure of the SCN.

## Visual Abstract

**Figure:**
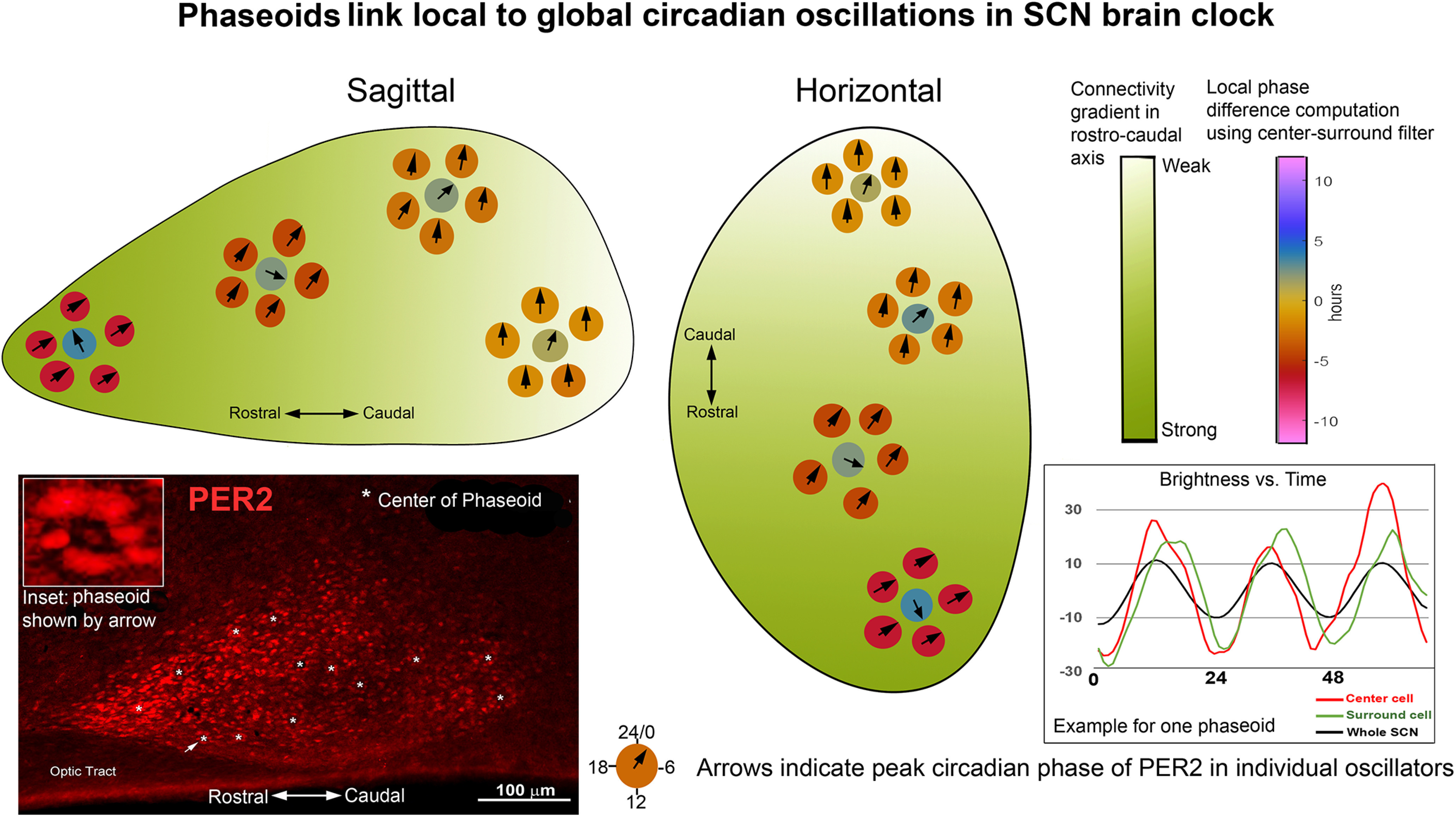

## Significance Statement

Oscillation is a fundamental property of information sensing and encoding in the brain. Using real-time imaging and modeling, we explore encoding of time by examining circadian oscillation in single neurons, small groups of neurons, and the entire nucleus, in the brain’s master: the suprachiasmatic nucleus. New insights into the network organization underlying circadian rhythmicity include the discovery of intermediate structures, termed “phaseoids,” characterized by groups of neurons which are stably out of phase with their neighbors. Modeling indicates that the pattern of phaseoids across the tissue encompasses a gradient in connectivity strength from the rostral to caudal aspects of the nucleus. Anisotropy in network organization emerges from comparisons of phaseoids and connectivity gradients in sagittal, horizontal, and coronal slices.

## Introduction

It is widely accepted that the phasing of neuronal oscillation is an important aspect of network organization and brain function (Buzsáki and Draguhn, 2004). The hypothalamic suprachiasmatic nuclei (SCNs) function as a master circadian clock that orchestrates circadian rhythms in behavior and physiology. Each SCN is made up of ∼10,000 neurons and the individual neurons contribute to circuits that support the coherent daily oscillation of the nucleus. While most SCN neurons express circadian oscillations, the individual cellular rhythms in the network are not synchronized in that they do not simultaneously reach peak phase (Schaap et al., 2003; Evans et al., 2011; Koinuma et al., 2013). Orchestration of stable circadian rhythmicity requires a network that couples individual SCN neurons to each other (Indic et al., 2007; Webb et al., 2009; Honma et al., 2012; Abel et al., 2016; Hastings et al., 2018; Tokuda et al., 2018; Wang et al., 2018; Finger and Kramer, 2020)

With respect to circadian timing, a challenge is to understand how coherent daily rhythms emerge in the brain master clock through interactions of its individual neurons, ensembles of neurons, and larger-scale oscillation of the SCN tissue as a whole. Substantial evidence indicates that stable phase differences occur not only between adjacent neurons (Quintero et al., 2003) but also among clusters of neurons in subregions of the nucleus (Yan and Okamura, 2002; Yamaguchi et al., 2003; Inagaki et al., 2007; Brown and Piggins, 2009; Evans et al., 2011; Foley et al., 2011; Pauls et al., 2014; Yoshikawa et al., 2017). While peak phase differs among neurons, relative phase does not drift (Yamaguchi et al., 2003), pointing to a non-uniform SCN network topology underlying tissue-wide oscillation.

Instead of synchronization of peak phase among individual elements, long-term, real-time luciferase reporter imaging of clock genes or proteins in SCN slices indicate phase waves that propagate over the entire nucleus with an ∼24-h rhythm. In coronal slices, these daily phase waves generally start in a distinct cluster of neurons in the arginine vasopressin (AVP)-rich dorsal or dorsomedial region of the nucleus (Evans et al., 2011; Enoki et al., 2012). It is noteworthy that there are marked differences in oscillatory patterns among slices, likely because of inclusion of different network components included at the time of tissue harvesting. Within an individual slice however, the phase relationships of serial oscillatory waves are stable if the tissue is not perturbed (Foley et al., 2011). An important question is how these phase patterns link to the underlying fixed aspects of the SCN network. The localization of major clusters of SCN peptides do not fully explain the patterns of oscillation (Evans et al., 2011), and the precise topology of the SCN connectome has been difficult to establish in part because of the small size, dense packing and heterogeneity of its neurons and the fine caliber of fibers (Van den Pol, 1980).

While understanding of the intra-SCN connectome is incomplete, the functional significance of the connections between two major regions, namely the ventral core and dorsal shell are well established (for review, see Honma, 2018). The core-shell framework has produced both biological and modeling work that provides substantial insight into SCN oscillation (for review, see Pauls et al., 2016). An aspect of network topology that escapes notice in studies of core-shell relationships is the possibility that SCN networks are anisotropic and that key aspects of network topology are lost following transection of fibers that course rostro-caudally. Studies of other oscillatory networks, such as the thalamus, highlight the principle that network oscillatory properties differ when brain sections are cut in the transverse versus the longitudinal axes (Gloveli et al., 2005).

The goal of this study was to determine whether fixed properties of SCN tissue, specifically those set by the localization and connectivity of its neurons might underlie the observed SCN oscillatory phase patterns and their variations. How the intact SCN’s anatomy, morphology, and connectivity gives rise to the phase relationships among SCN neurons or clusters of neurons remains elusive. Also elusive is how circadian oscillation is retained following ablation of major components of the nucleus (Rusak, 1977; LeSauter and Silver, 1999). To address some of the caveats in our understanding, we pair detailed morphologic analyses of fixed tissue, studies of real-time imaging of PER2::LUC expression in cultured SCN tissue and mathematical and statistical tools to explore SCN networks. We define the biological aspects of SCN organization that underlie the topography of individual cellular oscillations and investigate the impact of that evidence on simulations with a mathematical model. The biological results point to novel intermediate structures that we term “phaseoids.” Sagittal and horizontal slice orientations that maintain the SCN’s rostral-caudal axis reveal a global phase gradient associated with the magnitude of the local phase differences within the phaseoids. Modeling results show that a gradient of connectivity strengths between neurons can account for the observed phase gradient of the phaseoids along the rostral-caudal axis.

## Materials and Methods

### Visualization of SCN peptides in sagittal, coronal, and horizontal planes

To visualize the distribution of peptidergic cell types in the SCN, sections were stained for mENK, gastrin-releasing peptide (GRP), calretinin, glial fibrillary acidic protein (GFAP), DAPI, and AVP and vasoactive intestinal polypeptide (VIP; the latter in colchicine treated mice) using the material and protocols previously reported in (Varadarajan et al., 2018). To create the schematic, the localization of peptides and DAPI was based on representative sections at the largest extent of the nucleus in each plane.

### Oscillation criteria

As PER2::LUC expression in sagittal sections has previously never been described, we asked whether circadian oscillation is seen in all slices harvested or alternatively, whether it is restricted to the slices that bore core and shell components. To this end, each slice was assessed to classify oscillation, independently by two observers. In addition, slices were evaluated using Fourier analysis to determine statistical significance of the 24-h period.

### SCN slice culture, bioluminescence

#### Slice culture

Mice were decapitated and enucleated after cervical dislocation between zeitgeber time (ZT)5 and ZT9. The brain was removed and chilled in ice cold HBSS, followed by slicing of tissue (microslicer; Dosaka EM) at 100 μm in a sagittal, coronal, or horizontal plane. The brain slices were cultured on a membrane (Millicell-CM membrane, Millipore) with 1.3 ml of DMEM containing 0.2 mm D-luciferin K and 5% culture supplements as in Yoshikawa et al. (2013).

#### Bioluminescence recording

Images were obtained using a CCD camera cooled to −80°C (ImagEM, Hamamatsu Photonics; iXon3, Andor). Bioluminescence was recorded hourly for six consecutive days, starting immediately after decapitation. At the end of the recording period, the brain slices were fixed with 4% paraformaldehyde in 0.1 m phosphate buffer and prepared for immunohistochemistry. All procedures were approved by the Animal Research Committee of Hokkaido University.

### Visualization and analysis of luciferase in serial frames of the image stacks

The raw data consists of sequential images recording PER2::LUC expression over 1-h intervals. Data processing includes restricting the images to a region of interest thereby delineating the SCN and windowing the time series to a range in which movement of the tissue is minimal. Further, image sequences were restricted from the first frame without movement to the longest possible multiple of 24 h to alleviate artifacts in Fourier analyses. Raw images were imported into ImageJ (version 1.52). For visualization purposes, outliers were removed using manual observation of the histogram. The image was then imported into Photoshop, converted into RGB scale and a color gradient was applied. Next, the first peak of PER2::LUC expression time and each subsequent 3-h interval was captured for a total of a 27-h cycle. The brightness time series was computed as follows: (1) the data were restricted to the spatial ROI delineating the SCN, and the temporal ROI with minimal tissue movement; (2) the whole of the remaining data were then z-scored and plotted with the mean of each frame.

Only those with a robust 24-h period were considered for further analysis. A further screening was then done using mean brightness time series, to ensure that there were several complete circadian oscillations in each slice. All slices meeting these criteria were further analyzed. We focused on sections that contained the rostral and caudal poles, as these preserved the full rostro-caudal extent of the nucleus, and included the rostral and caudal poles that cannot be studied in coronal sections. Two slices were chosen in each orientation to illustrate the results.

### Global scale phase map: extracting the phase associated to the component of the signal with a 24-h period

For each pixel, we computed a discrete Fourier transform of the time series (described above as a multiple of 24 h). This results in each pixel having a complex number, α+iβ,associated to the component of signal with a 24-h period, which allows us to compute the phase:

ϕ=arctan(β/α).

Each phase is given in radians, which we can convert to hours: $\bar{\phi }=\frac{\phi }{2\pi }\cdot 24.$ As phase is a relative statistic, it can only be measured against a baseline, we normalize the phases across the SCN so that a phase of zero corresponds with the mean signal across the SCN at the period of 24 h. This results in the phase of every pixel being at most 12-h phase advanced, or 12-h phase delayed relative to the mean oscillation. The product of this process is a matrix P that aligns with the images in the frames of the PER2::LUC movies: P(x,y)is the phase extracted from the time series associated to the pixel in the (x,y) coordinate in a frame of the movie. Each lobe of the SCN was analyzed separately, and for visualization purposes, one of the two lobes was chosen for each of the horizontal and coronal slices.

### Computing local scale phase difference with a center-surround filter

Examination of the global phase maps raises the question of the extent of heterogeneous phases in localized patches of the SCN. To focus on this local analysis, we compare the average of phases over a cell-sized disk of pixels to an average of those of an annular region surrounding that disk. This is done by convolving a filter isolating each putative cell-like region with the matrix of surrounding phase estimates. We use a binarized difference of Gaussians filter to facilitate the computation. Such a filter is also called a center-surround filter, as it is positive on central disk and negative on a surround annulus.

We define the filter by

$$F\left(x,y\right)=\frac{1}{2\pi \sigma^{2}}e^{-\frac{x^{2}+y^{2}}{2\sigma^{2}}}-\frac{1}{2\pi K^{2}\sigma^{2}}e^{-\frac{x^{2}+y^{2}}{2K^{2}\sigma^{2}}},$$

where (x,y) are the coordinates in the image plane, and σ and K are parameters that delineate the center and the surround: the first term is a Gaussian with standard deviation σ, defining the center, and the second a Gaussian with standard deviation Kσ, which defines the surround. For our purposes, using σ=1,K=2 creates a filter which is positive on a central disk of radius four pixels (∼9 μm) and a surrounding annulus with outer radius 13 pixels (∼20 μm) where the filter in negative. These sizes are consistent with comparing neurons to the surrounding tissue in our data, as SCN neuronal radii are 4–4.5 pixels. Further, we binarize and normalize this filter by first replacing all values with absolute value less than 10^–5^ with zero. Then we replace the remaining positive values with +1 and negative values with –1. Last, we normalize the negative values of the filter by dividing by the number of pixels with a negative value and the positive values similarly. We denote the resulting filter by $\bar{F}\left(x,y\right)$. The circular annulus around a cell-like center we chose is based not only on patterns observed in the tissue but also because it is a general filtering approach used as a preprocessing technique for such problems as edge detection (Canny, 1986).

### Convolution of the center-surround kernel over the SCN

Convolving with this filter provides the difference in average phases between the disk and surrounding annulus centered at each pixel in the image. Applying center-surround filter to the SCN using 2D-convolution, $D=P⋆\bar{F}$, measures phase difference in the time series between each local disk and its nearest neighbors. The entry D(x,y) gives the difference between the average of the phases over the central disk of the filter, translated to be itself centered at the coordinates (x,y), and the average of the phases over the annular part of the filter.

Before implementing this method, we benchmarked the algorithm by evaluating whether it detected the visually identified cell-like region. In looking at the PER2 expression movies, we noticed roughly circular arrangements of PER2::LUC neurons that we could visually detect because of brightness differences with the surrounding tissue (also seen in fixed tissue). These circular arrangements were most obvious to the eye in the hours around the trough of the oscillation, at times of overall low PER2::LUC expression; they could not be seen at high points of PER2::LUC when the entire nucleus was bright and individual neurons could not be visualized by eye. We then compared the local phase difference results over the same region to assess whether the two methods matched. This benchmarking procedure confirmed that visually identified phaseoids, seen over several oscillations in the PER2::LUC movies, were detected by the application of the center-surround filter.

This benchmarking procedure confirmed that visually identified phaseoids, seen over several oscillations in the PER2::LUC movies, were detected by the application of the center-surround filter by merging the PER2::LUC intensity image with the local phase difference results. Photoshop was used to create the blended image shown in Extended Data Figure 3-1.

### Kuramoto coupling model

We use Kuramoto model systems to investigate the possible contribution of connection strength to the existence of the rostral-caudal gradient in the local phase maps. Kuramoto systems with cluster synchronization, where smaller clusters of oscillators synchronize to different phases, exhibit higher intracluster and lower intercluster connectivity (Favaretto et al., 2017) suggesting similar features might hold for the clusters of tissue we observe in the local phase maps. The Kuramoto model systems comprise a set of oscillators that are connected to and influence one another. Each oscillator in the system represents a neuron and is characterized by its intrinsic frequency, $\omega_{i}$, and the strength of its connections to other oscillators, $\left\{a_{i1},a_{i2},...,a_{in}\right\}$. We represent the model system by a set of n differential equations:

$$\dot{\theta_{i}}=\omega_{i}+K\overset{n}{\underset{j=1}{\sum }}a_{ij}sin\left(\theta_{j}-\theta_{i}\right).$$

Here, K is a global underlying coupling strength. Using the Runge–Kutta method, we can numerically solve this set of equations for the $\left\{\theta_{i}\right\}$, allowing us to test different hypotheses. To look at the contribution of coupling to the rostral-caudal gradient, we set up a simple testing framework. First, oscillators are arranged on a grid and connected to their nearest neighbors. Second, we vary the strength of the connectivity in one direction to evaluate the effects of strength on the patterns of the resulting phases of the oscillators. To formalize this, we construct a model using a 20 × 20 grid of oscillators arranged on a planar lattice. We set the intrinsic frequencies to be the same, $\omega_{i}\equiv 2\pi /24,$and K=5. Letting (x(i),y(i)) be the planar coordinates of oscillator *i,* we define $a_{ij}={\left(\frac{x\left(i\right)}{20}\right)}^{2}$ whenever *i* and *j* are neighboring oscillators in the plane (i.e., max{|x(i)−x(j)|,|y(i)−y(j)|}}=1). This change in strength across the rectangle of oscillators models weaker connection strength on one side of the SCN that strengthens as we move across the tissue to the other side. We solve numerically over a 240-h period, in steps of 1 min after providing initial conditions that are picked uniformly at random from [−π,π).

## Results

### Visualization of SCN in three planes

**Figure 1.**
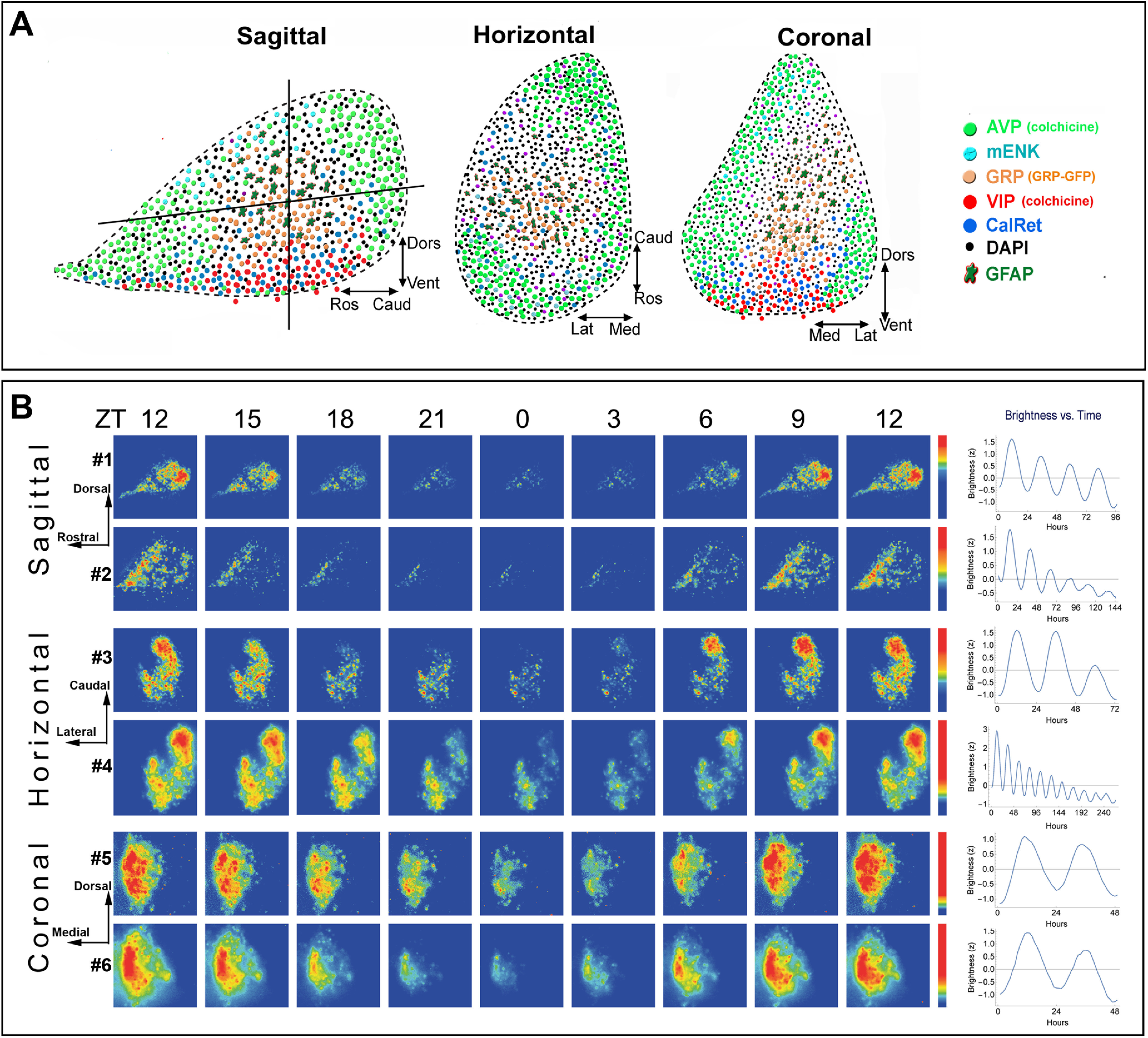
Architecture and PER2 expression of the SCN and bioluminescence heat maps and brightness time series. ***A***, Depiction of the peptidergic architecture by analysis of single-labeled SCN peptides in sagittal, horizontal, and coronal planes. The vertical and horizontal lines in the sagittal section indicates the plane shown in the adjacent cartoons of horizontal and coronal sections. DAPI label represents cells that are not positive for any of the markers used. VIP-containing neurons lie in the ventral core area. AVP neurons lie in the rostral protrusions and in much but not all of the outer borders of the nucleus. A GRP-rich area, along with nearby GFAP-positive elements, lies between the VIP core and AVP shell. In horizontal sections, the precise peptidergic content of an SCN slice differs markedly depending on the angle and depth at which the SCN is cut; if the ventral aspect is included in a slice, then both the core and shell are represented. In coronal sections, the localization of core (VIP- and GRP-rich) and shell (AVP-rich) regions are seen. ***B***, The spatiotemporal pattern in PER2::LUC bioluminescence in SCN slices is shown at 3-h intervals for representative sagittal, coronal, and horizontal slices. Time zero was defined as the time point with the lowest bioluminescent intensity (for details, see Materials and Methods). The pseudocolored images are normalized to the brightest image of each slice. The rainbow scale (blue, low and red, high expression) for each slice lies on the right side of the last panel. The SCN slices are numbered consistently to correspond on all figures. All slices were recorded for the same 6-d duration, starting immediately after harvesting the tissue. Mean circadian oscillation used in further analysis is shown in the right column. Explanation of differences among slices in the durations of data used for analysis is provided in Materials and Methods. PER2 expression in perfused tissue from animals killed at controlled times of day is shown in Extended Data Figure 1-1 and further information on methods is given in (Riddle et al., 2017).

The SCN is made up of a heterogeneous population of neurons. To set the stage for understanding the relationship of regionally specific clusters of cell types to the SCN network topology, we first mapped the peptidergic organization of the mouse SCN, delineating the major peptidergic cell types that can be retained when SCN tissue is prepared in sagittal, horizontal or coronal orientations (Fig. 1*A*). The SCN is a bilateral structure, lying on each side of the third ventricle and extending ∼350 μm dorsoventrally, 300 μm laterally from the third ventricle and 700–750 μm rostro-caudally (including a finger-like rostral projection). The full rostro-caudal extent of the SCN is best seen in sagittal sections. The distribution of these key peptides aligns fully with the spatial distribution of corresponding genes (Wen et al., 2020). The peptide maps in Figure 1 emphasize that the specific SCN neurons and networks captured when tissue is sectioned for *ex vivo* real-time imaging of oscillation can differ markedly depending on the precise tilt of the brain when it is blocked and on the orientation in which it is sliced. These differences among slices provoke the question of which cellular and network components are necessary for oscillation and whether anisotropy (directionality) is a determinant of the pattern of SCN oscillation.

10.1523/ENEURO.0078-21.2021.f1-1 Extended data Figure 1-1 PER2 expression at 2-h intervals in medial, mid and lateral SCN. To localize changes in PER2 expression so as to have a baseline against which to compare the *ex vivo* sagittal slices in the real-time imaging experiments, we assessed expression of the protein through the entire SCN in fixed tissue at 2-h intervals. Female (*N* = 11) and male (*N* = 25) mice were perfused and brains were processed to stain for PER2 (as in Riddle et al., 2017; rabbit anti-PER2 antibody used at 1:500; catalog #AB2202; RRID:AB_1587380, EMD Millipore Corporation). Sagittal sections of the SCN allowed visualization of the full rostro-caudal extent of the nucleus. Each dot represents the number and location of the PER2 nuclei observed in two to three brains at each time point. PER2-positive neurons can be seen at the trough of PER2 at ZT24/ZT0 to ZT4 in the mid and lateral SCN in the mid SCN. The rostral SCN expresses PER2 from ZT4 to ZT22. The photomicrographs in row 4 and 8 show the mid SCN. The implication of regionally localized PER2 expression is that the observed network architectures will depend on the precise orientation of the slice. Maintaining the rostral and caudal poles of the SCN may preserve important circuit components that are lost in coronal sections. Download Figure 1-1, TIF file.

### Effect of slice orientation on oscillation

We compared the effect of transecting SCN networks in three orientations by examining oscillation of PER2::LUC in sagittal, coronal, and horizontal slices (Fig. 1*B*). Imaging of sagittal sections has not previously been reported. For this reason, we first examined whether the results seen in these slices correspond to data on PER2 expression in immunochemically stained sagittal sections harvested from animals killed at specific circadian time points (Extended Data Fig. 1-1). The results are confirmatory: the peak and trough oscillations are separated by ∼12 h, and the overall oscillation has a period of ∼24 h (see pseudocolored images of changes over time and quantification of the brightness time series in Fig. 1*B*, left and right, respectively). The oscillation of the caudal SCN is more marked than the rostral aspect, and bears a different phase. The results for oscillation in our coronal and horizontal sections are consistent with previous reports on real-time imaging of SCN slices (Evans et al., 2011; Yoshikawa et al., 2017).

For real-time imaging of the sagittal slices, we next investigated whether the anatomy of the slice, determined after imaging, impacted the production of oscillation or whether all slices oscillated regardless of which circuit elements were present in the tissue. The results indicate that slices bearing both AVP and VIP neurons had robust rhythmicity while those lacking these peptides were not rhythmic (Extended Data Fig. 2-2; for oscillation criteria, see Materials and Methods). Slices bearing a large number of AVP neurons but lacking VIP were not rhythmic, consistent with previous reports in coronal slices (LeSauter and Silver, 1999; Aton et al., 2005).

### Observing phaseoids using biology and mathematics

**Figure 2.**
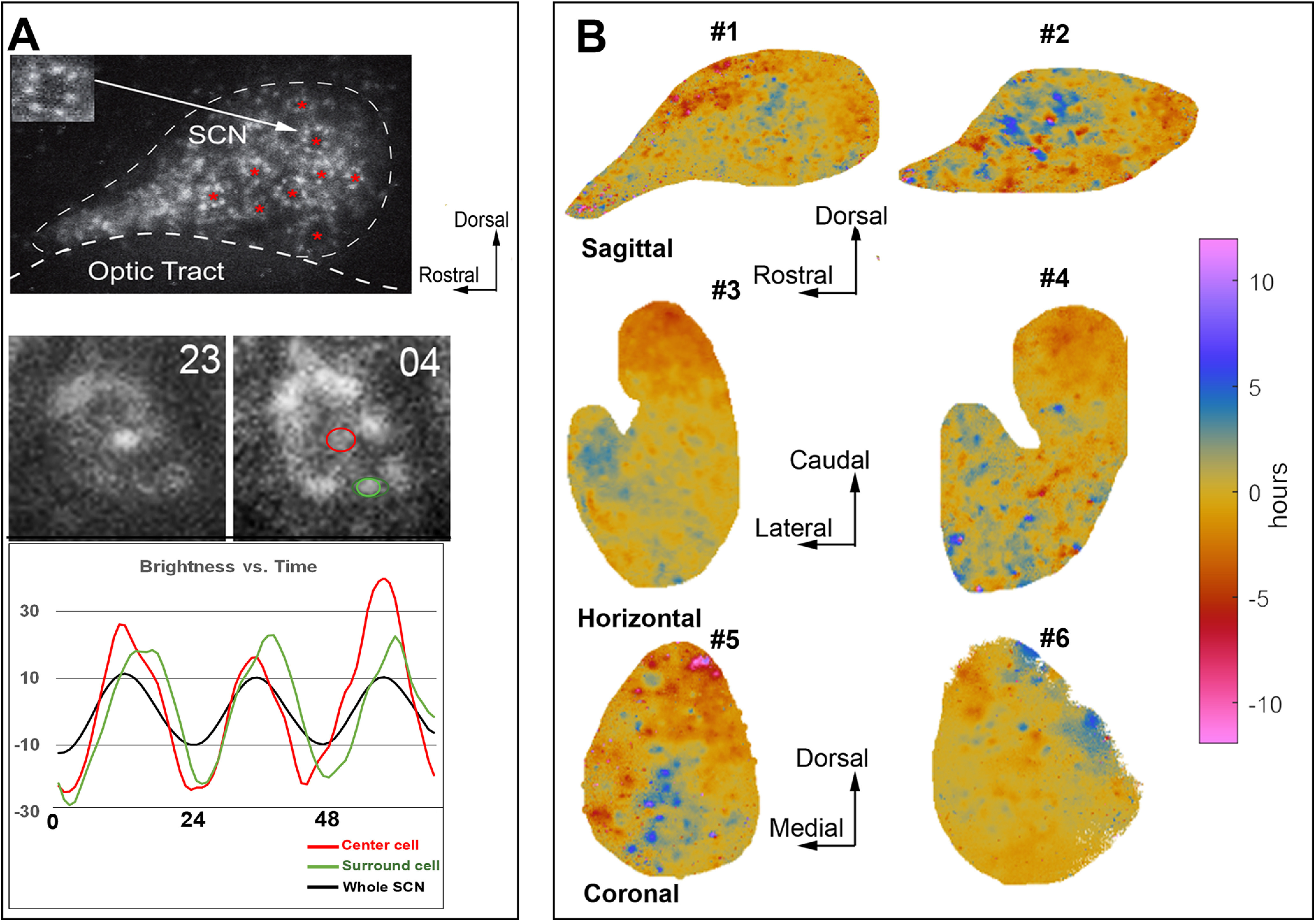
Global phase maps and phaseoids. ***A***, Phaseoids in the SCN. The top panel is a raw image of a bioluminescent recording near the trough of PER2::LUC expression in a sagittal section. Red asterisks highlight the location of phaseoids. The inset shows a magnification of the phaseoid indicated by the arrow. The middle panels are bioluminescent images of a phaseoid in a sagittal section taken at two time points. At time 23, PER2::LUC expression is higher in the center cell compared with its neighbors. Five hours later, PER2::LUC expression is higher in surrounding cells compared with the center cell. The bottom panel shows the PER2::LUC oscillation over ∼72 h in the center cell (red circle), surround cell (green circle), and the whole SCN for the phaseoid in the middle panel. ***B***, Mathematically assessed phase maps for sagittal, horizontal, and coronal slices of the SCN. Phase is represented by color, ranging from regions that are phase advanced (red) or phase delayed (blue) with respect to the tissue mean (yellow). The color of the 12-h advanced regions matches to those with 12-h delayed regions, as these will be in phase with one another in a 24-h oscillation. All sections exhibit areas that are phase advanced and others that are phase delayed often intermingled with one another. The analysis of immunohistochemical staining of SCN slices after imaging is shown in Extended Data Figure 2-1 using methods previously reported in (Yoshikawa et al., 2015). Phaseoids in fixed SCN tissue are shown in Extended Data Figure 2-2.

In the real-time imaging studies, close examination of the tissue near the trough of the oscillation reveals local phase heterogeneity with small populations of cells substantially out of phase with the surrounding tissue (Fig. 2*A*, top panel). We designate these groupings as intermediate structures, here termed phaseoids. Phaseoids (denoted by red asterisks) are a local group of cells with stable phase heterogeneity, in which a cell is surrounded by a group of cells out of phase with it. A representative phaseoid from the imaged material is shown at two time points with a phase difference of ∼5 h between the center cell and the surrounding cells (Fig. 2*A*, middle and bottom panels). These phaseoids are not an artifact of the preparation, as they can also be seen in SCN sections from animals killed near the trough of PER2 protein expression.

10.1523/ENEURO.0078-21.2021.f2-1 Extended data Figure 2-1 Postimaging immunohistochemical analysis of histology. After imaging, sagittal slices (*n* = 22 slices, 100 μm, from 6 mice) were fixed with 4% PFA after the bioluminescence recording, and immunohistochemically labeled with a cocktail of antibodies against AVP (AVP-NP, PS419) and VIP (Peptide Institute, 14110). Immunohistochemical staining was examined by fluorescent microscopy (BZ9000; Keyence) as previously reported (Yoshikawa et al., 2015). ***A***, Schematic drawing of the SCN. The blue lines indicate the plane of 100-μm sagittal slices that were made. Numbers (–2 ∼ +2) indicate position of the sagittal slices with respect to the midline. ***B***, Images of bioluminescence, immunohistochemical staining for AVP, VIP, and overlay of AVP and VIP. ***C***, Chart comparing robustness of oscillation and expression of AVP and VIP in each slice. Slice IDs indicate good (black) and poor (grey) rhythms. Number of immunopositive cell bodies for VIP and AVP are expressed in symbols. ++, high; +, medium; ±, low; –, none. Robust oscillation requires both AVP and VIP expression. Slices bearing a large number of AVP neurons but lacking VIP showed poor rhythm. The one slice that had both peptides could not classified with respect to rhythmicity due to technical equipment problems. Anatomical analysis of peptide expression was conducted independently and prior to classification of rhythmicity. The number of positive cell bodies were scored as follows. For VIP: >10 cells = ++, 5–9 cells = +, 1–4 cells = ±, no cells = –. For AVP: >20 cells = ++, 5–20 cells = +, 1–5 cells = ±, no cell = –. Download Figure 2-1, TIF file.

10.1523/ENEURO.0078-21.2021.f2-2 Extended data Figure 2-2 Phaseomes in fixed SCN tissue: photomicrograph of a 50-μm sagittal SCN section immunostained for PER2 (red) at ZT20. White asterisks show the location of phaseomes. The inset is a magnification of the phaseome indicated by the white arrow. Slices were processed for immunocytochemistry as in Riddle et al. (2017). Download Figure 2-2, TIF file.

### Relation of global to local phase

To explore global and local phase gradients through the full extent of the SCN, we devised a general analytical tool and applied it across different slice orientations. For global phase gradients, the phase of each pixel was assessed against the mean phase of the tissue (the global phase). This allowed determination of the effects on oscillation of preserving limited aspects of the network. Analysis of the temporal pattern of PER2::LUC expression in the slices through Fourier methods allowed identification of the phase of oscillation of any region of the SCN tissue relative to the mean circadian oscillation of the tissue as a whole (see Materials and Methods). Using such methods, we investigated whether phase maps differ by slice orientation and found that global phase maps show systematic phase anisotropy and heterogeneity (Fig. 2*B*).

Phase is represented by color, ranging from small regions that are phase advanced (red) or phase delayed (blue) with respect to the tissue mean (yellow). In the sagittal slices, there are neurons at the rostral pole and those in an area adjacent to the core that are phase delayed (blue speckled areas) relative to the mean tissue oscillation. Similarly, the horizontal sections also show near anti-phase relations between the rostral and caudal aspects of the tissue, with the caudal area substantially phase advanced (∼5 h) and the rostral aspect of the tissue substantially phase delayed (also ∼5 h) relative to the mean. In coronal sections, we find, consistent with previous literature, a phase advanced region in the dorsal-medial aspect of the tissue. The rest of the phase map varies among slices (as previously reported; Foley et al., 2011; Pauls et al., 2014), likely because of heterogeneous sampling of the circuit depending on which part of the rostral-caudal extent of the slice was studied.

Phaseoids have not been previously reported but are consistent with prior work showing that adjacent neurons can be out of phase with each other (Quintero et al., 2003). Local phase behavior may be a common occurrence across the SCN but could differ in structure depending on slice orientations and may be obscured by slice heterogeneity. The large global phase differences across the tissue confounds a local analysis, phase gaps of, for example, 4 h might occur between adjacent regions with global phases at 10 and 6 h from the mean in one part of the slice, and between regions with phases at 0 and −4 h at another area. This consideration prompted us to develop a mathematical method to examine localized phase maps based on a center-surround filter that exposes relative local phase differences.

### Application of annular filter

**Figure 3.**
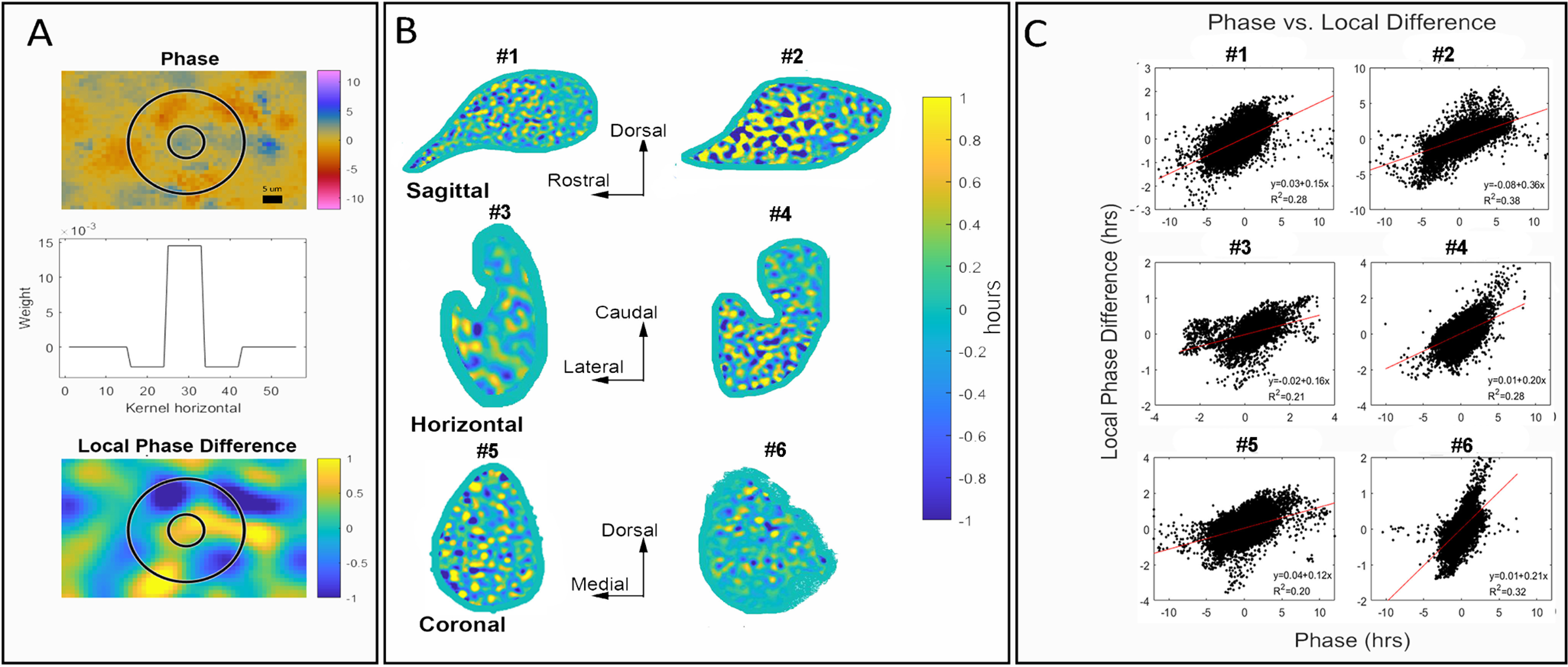
Local phase comparisons reveal phaseoids. ***A***, Center-surround filter is superimposed on a magnified view of a phase map. Upper panel, In the center, there is a cell-sized region that is phase leading the mean signal. In the annular region, several cell-sized areas are phase lagging the mean signal. This is consistent with evidence (Fig. 2) showing that neighboring areas have different levels of expression in similar local spatial arrangements. Middle panel, Cross-section of the center-surround filter used to compute the local phase differences. Lower panel, Results of the local phase difference computation on the same region and with the same center circle and surround annulus as the upper panel. The computation isolates the cell-sized regions and identifies them as phase leading or phase lagging the neighboring pixels, allowing a full slice analysis of local phase differences. The example shown here is the same phaseoid as that shown in Figure 2*A*. Note that the original observation of the phaseoid was by visual identification in the movie, and it is well captured by the filter (shown in Extended Data Fig. 3-1). ***B***, Local phase differences as computed using the center-surround filter show numerous portions of the SCN that are out of phase with their neighboring tissue. Sagittal and horizontal slices show a gradient along the rostro-caudal axis where phase differences are smaller at the caudal than the rostral aspect. The colors in this figure have been truncated to emphasize differences in values close to zero. The green border around the edge of each SCN is the result of “padding” to fill the kernel. ***C***, Plot of the global phase against the local phase difference to examine the relationship between the two. Each point represents a single pixel. On the horizontal axis (global phase estimate of the tissue), positive values indicate oscillation that lags the mean oscillation while negative values indicate leading the mean oscillation. Linear regression lines (red) have high statistical significance and support the hypothesis that areas with larger local differences are more strongly leading or lagging the mean.

Differences in phases can be difficult to detect by visual examination of individual still images. Visual identification of phaseoids best occurs for that subset in which PER2::LUC-expressing cells are active during a global trough. To enable phaseoid detection across the whole of the circadian oscillation, we constructed a center-surround filter to report the difference between the average phase in a cell-size disk of pixels around each point and that of a local annulus of equal radius around the disk (Fig. 3*A*, middle panel; for more details of center-surround filter and “kernel,” see Materials and Methods). The local phase analysis allows the examination of phaseoids computationally. This is illustrated by a very prominent phaseoid identified from a global phase map in Figure 2*B* that has a phase difference of ∼10 h. In a representative example, the center cell size disk (blue) phase-lags the mean oscillation by ∼5 h (Fig. 3*A*, top panel). Surrounding it are four neuron-sized regions (red) that lead the mean oscillation by ∼5 h.

10.1523/ENEURO.0078-21.2021.f3-1 Extended data Figure 3-1 This figure gives a comparison between manual identification of neurons that are possibly out of phase with their surroundings and the results of the local phase difference computations. The left panel shows a region of the SCN in a movie frame near the trough of the mean oscillation of the tissue. In observing the movie, we could identify several neurons (marked with a red asterisk) that seemed to be out of phase with much of the surrounding tissue. In the middle panel, different intensities of green indicate the results of the local phase computations, with the more negative phase differences shown in brighter green. The locations of the neurons from the first panel are marked with blue asterisks where the color scale indicates that they are oscillating between 2 and 3 h behind the surrounding annular region. The right panel shows a merging of the two other panels, demonstrating that the higher intensity areas from the first panel coincides with more negative local phase differences from the second panel. The overlay creates magenta asterisks showing that the placement of the images coincides. Download Figure 3-1, TIF file.

Convolving by the filter over each pixel of the phase map results in a local analysis (Fig. 3*A*, bottom panel). Phaseoids vary in the differences in the phase relationships of their components. The strength of the phaseoids vary across the tissue and we define the “strength” of a phaseoid as the magnitude of the difference between the mean phase of the center and the mean phase of the surround. For example, a weak phaseoid would have a center-surround phase gap <1 h in magnitude, while a strong one would be >1 h (like the pictured example in Fig. 3*A*, bottom panel). This computational approach extends our ability to detect phaseoids beyond those that are visually detectable because their surrounds are active in the trough of the oscillation.

### Center-surround analyses of local phase comparisons reveal phaseoids throughout the SCN

Identifying the phaseoids computationally allowed us to determine whether local phase organization was reliant on the direction of the slice and/or the part of the tissue that was sampled over any extent (Fig. 3*B*). This is particularly relevant because intermediate structures have not been identified previously. The results indicate that phaseoids exist regardless of the orientation of the slices, but that their strength differs depending on the direction of slicing and the extent of the SCN examined, particularly on the rostral-caudal axis. The local areas have average phases that differ by ∼1 h regardless of slice orientation. This is shown in Figure 3*B*, by the color in the convolved map, which shows areas that are advanced (blue) and delayed (yellow) relative to the surrounding tissue mean (green). Note that phase differences in the local calculation are smaller than in the global calculation because of averaging. Many pixels in the global phase map (on which the kernel is convolved) are near the mean phase (i.e., phase difference of 0) and when included in the local kernel this brings the average closer to zero. We truncated the color scale to emphasize the pattern of local differences, even when the phaseoids are weak. The brightest yellow and darkest blue regions can have local differences larger than one or less than minus one, respectively.

### The strength of phaseoids differs across the SCN’s rostral-caudal extent and among slice orientations

In the sagittal and horizontal sections, phaseoids are stronger close to the rostral pole and weaker near the caudal extent (Fig. 3*B*, top two rows). We see this visually in the extent of the green areas (representing tissue mean) between the phase advanced (blue) and delayed (yellow) elements. In coronal sections, where the rostro-caudal extent is limited, we do not detect a consistent gradient on either the dorsal-ventral or medial-lateral axis (Fig. 3*B*, bottom row). As indicated in Figure 1*A*, the precise components of SCN tissue and the specific network components that are included in a slice will depend on precisely how it is blocked and cut. The coronal sections transect all rostro-caudal connections while this is not the case for sagittal and horizontal slices.

### The magnitude of local phase differences is related to the global phase deviation

Comparing the global phase estimates of the tissue (horizontal axis) to the local phase differences (vertical axis) reveals an interesting relationship (Fig. 3*C*): areas that lead the mean oscillation by the largest amount tend to have large negative local phase differences, while those that lag by the largest amounts tend to have large positive local phase differences. Including the ordinary least squares regression line (red) reveals positive linear slopes in each case. These results are statistically significant, with *p* values estimated below machine tolerance in every case (all *p* < 10^–16^). If there were no relations between local phase differences and the global properties of the oscillation, we would expect no detectable correlation between global phase and local phase differences and the regression line would be horizontal. Instead, we see a positive correlation in each instance regardless of orientation of the tissue.

### Kuramoto models connect the magnitude of local phase differences with the strength of local connectivity

**Figure 4.**
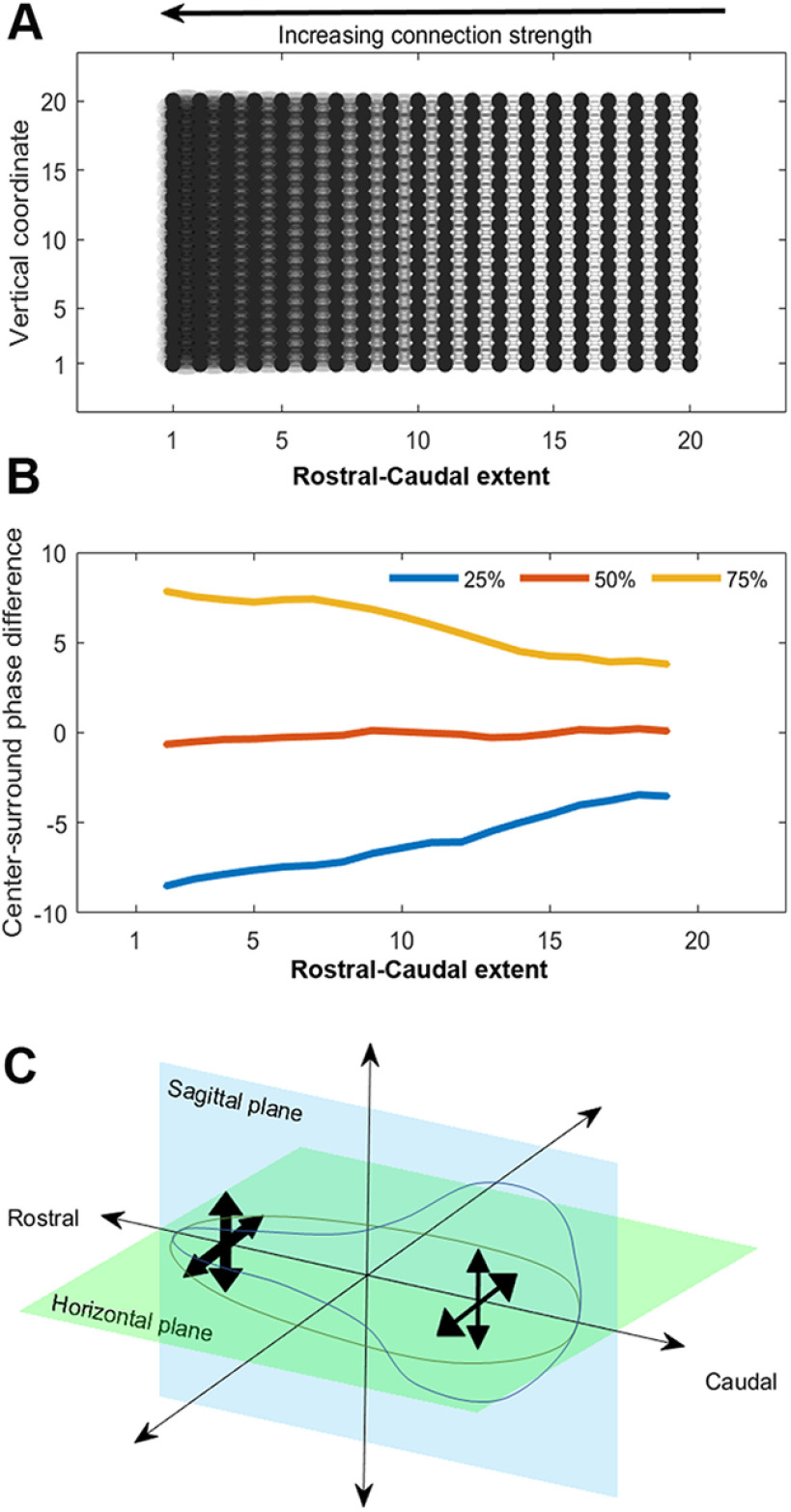
Kuramoto models connect the magnitude of local phase differences with the strength of local connectivity. ***A***, A schematic of the network connectivity we use in simulations using the Kuramoto coupled oscillator model. The differences in shading from right to left indicate quadratically growing strength of the nearest neighbor coupling in the model. ***B***, Results of applying the center-surround filter to simulated data over 500 trials. We report the quartiles of the distribution of local phase differences for each column of oscillators (as in ***A***) as we move from right to left, showing that as the connectivity grows, so does the magnitude of the local phase differences. ***C***, A schematic summarizing the interpretation of the local phase differences in the context of simulation results. Comparing to simulation results provides evidence that connection strength is weaker at the caudal edge of sagittal and horizontal sections and grows as we move toward the rostral tip.

We next asked what might be causing this rostral-caudal gradient. For non-chaotic systems of coupled oscillators, connection strength is intimately related to the phase dynamics of the system, leading us to hypothesize that the topology of the SCN connectome that is retained when slicing in different directions gives rise to the observed gradients in phaseoid strength. To test this hypothesis, we turn to mathematical modeling to examine the potential role of connectivity in the creation and strength of the local phase differences. We created a mathematical simulation encoding some of the properties of the SCN by constructing a 20 × 20 grid of oscillators with identical intrinsic frequencies that are linked to each of their four nearest neighbors. We change the connectivity by manipulating coupling strength, quadratically increasing it as we move across the grid from right to left horizontally (Fig. 4*A*). In this depiction, the coupling strengths of two oscillators in the same vertical column are identical, but oscillators in the same row have different strengths depending on their locations in the grid.

We solved the associated Kuramoto system numerically over a period of 240 h and calculated the phase of each oscillator (500 trials). We then applied a center-surround filter to the calculated phases, akin to the phaseoid detector described above (Fig. 3*A*). For each vertical column of oscillators, we consider the distribution of phaseoid strengths and compute their quartiles. Figure 4*B* reports these quartiles as a function of the horizontal coordinate of the oscillator within the grid. We compare size of the difference between the 75th percentile curve (yellow) and the 25th percentile curve (blue) to assess the strength of the phaseoids as a function of coupling strength. The difference between the lower and upper quartiles is more compact on the right-hand than the left-hand side, indicating that the strength of the phaseoids decreases along with the strength of coupling. These results provide evidence for the hypothesis that the greater the phaseoid strength, the stronger the local coupling between the oscillatory neurons (Fig. 4*C*).

## Discussion

### SCN networks as accessible models of oscillation

It is increasingly clear that systems in the brain responsible for temporal representation at many timescales rely on specific network organizations to sustain their activity (Buzsáki, 2006). Network oscillations can bias input selection, temporally link neurons into dynamic assemblies, and modulate synaptic plasticity. The SCN is a uniquely accessible empirical model to study oscillatory networks: it is self-contained, it controls behavior and is reflected in observable physiological responses throughout the body.

### What is new in this work

The present work further demonstrates that while individual neurons oscillate, the rhythm in the SCN relies on the specific elements that are present in the network as a whole: the tissue is the issue. The observed oscillation in real-time imaging of slices depends on what parts of the network are present after physical transection, and on the spatial and temporal resolution at which the tissue is being studied. Our use of biological, analytic and simulation tools demonstrate processes at multiple levels of analysis from individual cells to local and global organization in SCN networks and reveal the phaseoid as an intermediate, local unit of organization. While previous research has not identified local phaseoid-like structures in the SCN, the present findings are consistent with other locally identified neural units in the brain, such as the hypercolumns in area V1 (Hubel and Wiesel, 1968) and striosomes of the striatum (Graybiel and Ragsdale, 1978) that constitute intermediate structures parallel to the phaseoid. We have defined the strength of the phaseoid as the magnitude of phase difference between the center and surrounding annulus of phase-locked cells. We find that the strength of phaseoids varies in a systematic gradient across the tissue if the rostral-caudal axis is preserved. Interestingly, this gradient of local organization of phaseoids is aligned with a previously reported global phase gradient along the same axis in tissue harvested from animals held in long daylengths (Yoshikawa et al., 2013). The overall strength of the phaseoids is greater in the rostral aspect, which tends to phase lag, and smaller in the caudal aspect which tends to phase lead in sagittal and horizontal slices (Figs. 2*B*, 3*B*). Furthermore, strong phaseoids with negative local differences are associated with leading the global phase while strong phaseoids with positive differences lag it (Fig. 3*C*), which is to say the relationship between local phase differences and global phase is positive regardless of how the tissue is cut. The linkage between local and global organization highlights the potential functional role of the phaseoids in integrating local oscillatory information, phaseoids are not merely physical structures, but a mesoscale component of the machinery that allows the SCN to construct and maintain a robust and consultable circadian rhythm.

Our consideration of phaseoids, by the nature of the data, is necessarily two-dimensional but in the full three-dimensional structure of the SCN, the phaseoids may have more complex structures. The phaseoids we observe appear as rosettes. These structures are supported by observations made by eye in the PER2::LUC imaging movie frames, are also detected in fixed tissue, and are more fully revealed and characterized in the analytic tool we developed. In an intact SCN, the three-dimensional structures of the phaseoids may take many possible topological types, spheres, cylinders, tori (Harding et al., 2014).

We propose an impressively parsimonious model for the cause of these local/global patterns seen in the rostro-caudal gradient of phaseoid strength. The Kuramoto simulation results suggest that changes in strength of local coupling can produce similar patterns in model systems. Stronger local connectivity leads to stronger phaseoids in coupled oscillators (Fig. 4). It has been suggested that the brain clock network bears properties of small world networks (Strogatz, 2001; Vasalou et al., 2009; Hosseini and Kesler, 2013), which have tight local coupling alongside some longer-range connectivity. Our work helps to delineate possible structures for the local coupling, connecting it functionally to properties of the oscillation across the tissue.

### Relationship to prior work

The results are consistent with previous descriptions of phase in clusters of SCN neurons. The occurrence of individual neurons having elevated PER2 protein at the overall trough of expression have previously been reported: in prior work, however, it was not known whether these cells oscillate in antiphase with the larger population, or whether they are arrhythmic and continually express PER2 (Herzog et al., 1997; Hastings et al., 1999; Field et al., 2000; Nakamura et al., 2001; LeSauter et al., 2003). Our results indicate that an antiphase population is rhythmic, with high PER2 expression between ZT0 and ZT4 and low expression by ZT6 (Extended Data Fig. 1-1).

While phase dispersal and phase waves have been described, the occurrence of phaseoids or other intermediate structures has not been noted previously. Perhaps these were seen but not investigated or, alternatively, this may be because of slice thickness. Our slices in the real-time imaging preparations are thin (100 μm), allowing for better cellular resolution, while in many other laboratories, slice thickness is ∼300 μm. With 300-μm slices, it is difficult to visualize many individual cells simultaneously. Thin slices may have less of the global network than thick ones but optimize single cell analysis. Another factor may be the use of noise reduction algorithms or use of megapixels, which also reduce the resolution. Choices within these algorithms include decisions on how many pixels to use to smooth the signal and how to smooth the signals, which could have obscured phaseoids in prior reports.

### Importance of phase heterogeneity for timekeeping

Physiologic and behavioral functions, including feeding, drinking, sleep-wake, body temperature, hormonal rhythms, and enzyme activity, have circadian rhythms with specific circadian peaks. To accurately assess circadian time at every time of day requires consulting cells whose PER2 concentrations are changing over time. Phase heterogeneity in PER2 expression allows this precise consultation throughout the circadian cycle, because at any phase of the mean oscillation, some cells will have swiftly changing expression of PER2. While the present work focuses on expression of PER2, the principle of heterogeneity in cellular rhythms applies more generally to cellular activity of a variety of responses. The precise consultation throughout the circadian cycle is enabled because at any phase of the mean oscillation, some cells will have rhythms at a particular phase. Our findings imply, in addition to sequentially phased PER2 rhythms, phaseoids may enable even more precise and specific regulations in overt rhythms.

An analogous finding in the visual system is that the neurons that provide the most information about the orientation of an edge are those whose firing rates change the most, rather than those that fire the most when presented with similarly oriented direction of motion (Osborne et al., 2004). Protein concentrations of the mean signal in the SCN overall change slowly, especially when they are near the peak or trough of expression. Knowing the mean expression level of PER2 gives only a rough time signal, whether it is near the peak, trough, or in between. Higher precision requires information complementary to mean concentration: the rate of change in concentrations of cells out of phase with the mean. Rapid changes in concentration within phasically heterogeneous cells provide continuously accurate time of day information regardless of the state of the mean oscillation. The advantages of this heterogeneity likely represent a general property of information encoding in the brain.

## Synthesis

Reviewing Editor: Douglas Bayliss, University of Virginia School of Medicine Decisions are customarily a result of the Reviewing Editor and the peer reviewers coming together and discussing their recommendations until a consensus is reached. When revisions are invited, a fact-based synthesis statement explaining their decision and outlining what is needed to prepare a revision will be listed below. The following reviewer(s) agreed to reveal their identity: Karen Gamble, Yasufumi Shigeyoshi.

This manuscript was assessed by two reviewers who found that the work describes an interesting and novel characteristic of activity - so-called “phaseomes” that display stable local phase differences within the SCN. You will see that the reviewers have identified a number of important modifications for clarity, including suggesting that you reconsider the “phaseome” terminology. Some additional analysis has also been recommended, most notably further validation of the center-surround filter and some experiments to test the interpretation that differences in connectivity account for the delineation of individual phaseomes. These issues should be addressed in a revised manuscript.

The original reviewer comments are provided below.

Reviewer #1

Advances the Field

This is a very interesting and deep analysis of the phase network and organization within the brain’s primary circadian pacemaker in the SCN - which is well known for having the most robust circadian network in the mammalian system. Despite this recognition, our understanding of the network properties that make this oscillation persist for so many cycles is in its infancy. Here, the researchers have used real-time imaging of PER2::LUC across different anatomical orientations to explore how oscillating neurons influence the rhythms of adjacent neurons, discovering a center-surround network (“phaseomes”) which are further explored by computational modeling. The work is novel and insightful and is likely to spur further studies in this area. Other labs can use these methods to investigate phaseomes in other contexts and other oscillating systems.

Visual Abstract

The visual abstract succinctly describes the take-home findings of this study which is technically complicated.

Extended Data

Code does not appear to be provided.

Comments

This is a very interesting and deep analysis of the phase network and organization within the brain’s primary circadian pacemaker in the SCN - which is well known for having the most robust circadian network in the mammalian system. Despite this recognition, our understanding of the network properties that make this oscillation persist for so many cycles is in its infancy. Here, the researchers have used real-time imaging of PER2::LUC across different anatomical orientations to explore how oscillating neurons influence the rhythms of adjacent neurons, discovering a center-surround network (“phaseomes”) which are further explored by computational modeling. The work is novel and insightful and is likely to spur further studies in this area. Other labs can use these methods to investigate phaseomes in other contexts and other oscillating systems. Only minor comments are listed below to improve this body of work:

1. Line 264: Add references to prior work in the SCN using sagittal sections acknowledging that this studies did not use real-time imaging.

2. Line 292-293: DAPI could also stain for non-neuronal cells?

3. Figure 1B, right column: Having different scales for the Y axis (number of cycles) is distracting. It would be best to show the 27 hours used for analysis (in most cases?) as the same for all and then an additional extended axis for those rhythms beyond 27 hours. Along these same lines, In extended data Fig 2, was the rhythmicity impacted by the number of cycles that were rhythmic in each culture? Were slices at 0, [1], or [2] levels more or less likely to have several cycles of data?

4. Reference: In several, places the Riddle reference is highlighted but not included in the reference list.

5. Extended Data Fig 2-2: this section is at 50um thickness - was this for the post-staining only or was the imaging for his culture also done on 50um thick sections? Also there is a typo: “wer"

6. In Extended Data Fig 1-1, there is a clear anti-phased PER2 group of cells in the middle of the “Mid” sections - did this location of the SCN have more/less or stronger/weaker phaseomes?

7. Given the utility of this approach for other labs and applications, the authors should provide as much detail as possible in the analytical and imaging techniques used so others can also examine “phaseomes” in other contexts.

8. The computational modeling provided would be stronger if compared to other filters besides center-surround to show that this filter is the best fit.

Reviewer #2

Advances the Field

The authors found a new structure “phaseome” in the SCN, the center of the mammalian circadian center. The importance of the structure has not been clarified but the newly found structure may provide new insights on the mechanism of the circadian rhythm.

Visual Abstract

Good to show the structure of the phaseome with phases shown in the neurons and the localization is also shown.

Comments

The authors found, in the SCN, small local groupings of neurons that form ringed structures, here termed “phaseomes” showing stable local phase differences. It is the first time that such a structure has been clarified, and it is highly possible that it contributes to the function of SCN. The authors are exploring its functional significance by exploring phaseome.

I have some concerns.

Major concerns;

#By examining the phase of the central cells in Phaseome, they draw the conclusion that there is a difference in connectivity, and perform numerical simulation to show the applicability of that hypothesis. However, I do not understand why they conclude that there is a difference in connectivity by only analyzing the phase difference in the phaseomes with state of synchrony. The cell types that make phaseome may differ region by region in the SCN. It is possible that the coupling of cells is stable with a fixed phase difference. Therefore, It is difficult to connect the consistency of phase with the strength of coupling. The strength of the coupling can be examined by decreasing the connection among oscillating neurons by adding small amount of TTX, MDL, FK and observe which regions lose the synchronization.

#The authors searched for phaseome using a center-surround filter but I am not sure the filter worked well to detect the phaseome. The sensitivity and specificity of this filter should be shown. The author should compare it with the visually recognized image of phaseome. That is, when this formula is used, the sensitivity and specificity should be described by comparing with the phaseome visually recognized. Also, please describe how many phaseomes were present in each slice.

#I wonder why they call the ringed structure “phaseome”? Proteome, transcriptiome, and metabolome indicated the complete set of expressed proteins, transcripts and metabolic substances. So the impression of phaseome is that all set of the phases expressed in the oscillating neurons in the SCN. But in this case, the word “phaseome” indicates ring-shaped structures in the SCNwith phase difference by the centered neurons and surrounding neurons. The authors should replace the word “phaseome” with another word to avoid misunderstanding.

Minor concerns;

#254 the specific SCN neurons and networks captured when tissue is sectioned for ex vivo

255 real time imaging of oscillation can differ markedly depending on the precise tilt of the

256 brain when it is blocked and on the orientation in which it is sliced.

The relationship between the localization of peptides and the localization of phaseome should be described in the manuscript.

#387 Phaseomes vary in the differences in the

386 phase relationships of their components. The strength of the phaseomes vary across

387 the tissue and we define the ‘strength’ of a phaseome as the magnitude of the phase

388 gap between the center and surrounding cell-like regions.

The word “strength” used in the above sentences is ambiguous. What is the difference between the magnitude and strength. The author should clearify the difference between magnitude and strength. In addition, it is necessary to explain the significance to define the word “strength”.

#196 Figure 5A shows the shape of the center-surround filter

197 superimposed on the phase maps of the tissue.

I cannot find the Figure 5A.

#238 as we move across the tissue to the other side. Figure 6A shows a schematic of this

239 setup.

I cannot find the Figure 6A.
