# Supplementary figures and images for: Phase Gradients and Anisotropy of the Suprachiasmatic Network: Discovery of Phaseoids
Source: eNeuro. 2021 Sep 8;8(5):ENEURO.0078-21.2021. doi: 10.1523/ENEURO.0078-21.2021 (PMC8431825; doi:10.1523/ENEURO.0078-21.2021)

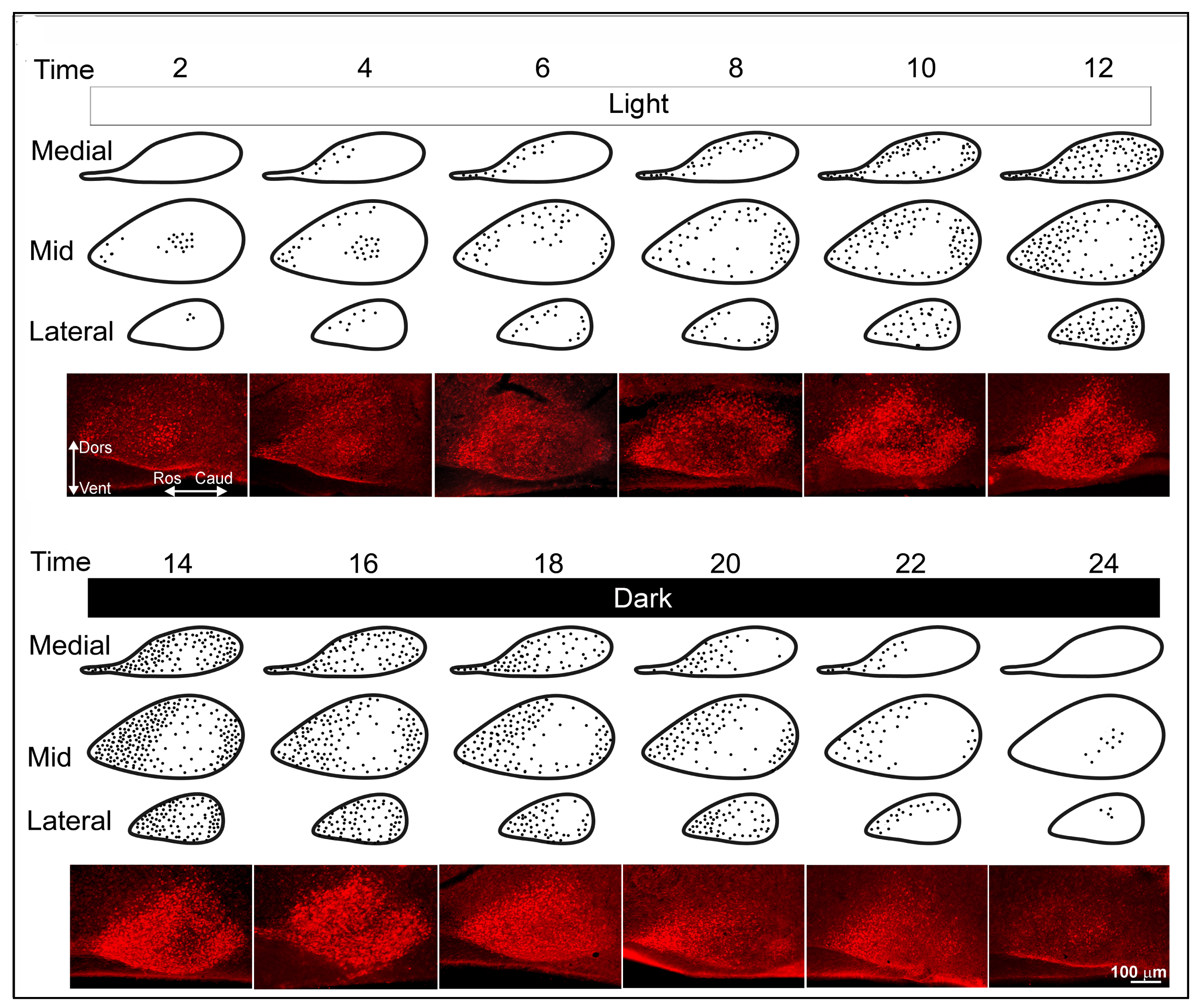

Supplement: Extended data Figure 1-1 — PER2 expression at 2-h intervals in medial, mid and lateral SCN. To localize changes in PER2 expression so as to have a baseline against which to compare the ex vivo sagittal slices in the real-time imaging experiments, we assessed expression of the protein through the entire SCN in fixed tissue at 2-h intervals. Female (N = 11) and male (N = 25) mice were perfused and brains were processed to stain for PER2 (as in Riddle et al., 2017; rabbit anti-PER2 antibody used at 1:500; catalog #AB2202; RRID:AB_1587380, EMD Millipore Corporation). Sagittal sections of the SCN allowed visualization of the full rostro-caudal extent of the nucleus. Each dot represents the number and location of the PER2 nuclei observed in two to three brains at each time point. PER2-positive neurons can be seen at the trough of PER2 at ZT24/ZT0 to ZT4 in the mid and lateral SCN in the mid SCN. The rostral SCN expresses PER2 from ZT4 to ZT22. The photomicrographs in row 4 and 8 show the mid SCN. The implication of regionally localized PER2 expression is that the observed network architectures will depend on the precise orientation of the slice. Maintaining the rostral and caudal poles of the SCN may preserve important circuit components that are lost in coronal sections. Download Figure 1-1, TIF file. [file enu-eN-NWR-0078-21-s03.tif]

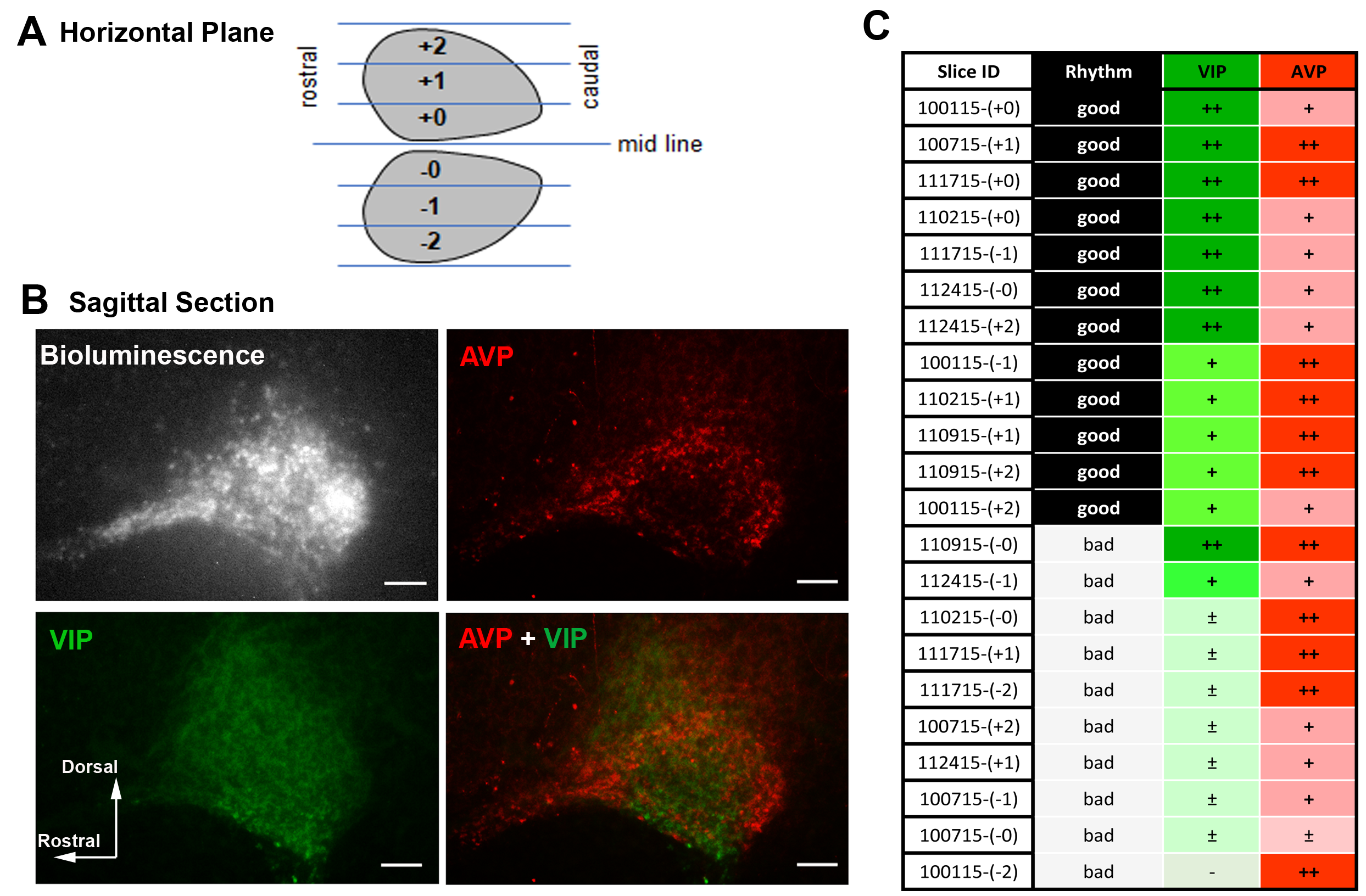

Supplement: Extended data Figure 2-1 — Postimaging immunohistochemical analysis of histology. After imaging, sagittal slices (n = 22 slices, 100 μm, from 6 mice) were fixed with 4% PFA after the bioluminescence recording, and immunohistochemically labeled with a cocktail of antibodies against AVP (AVP-NP, PS419) and VIP (Peptide Institute, 14110). Immunohistochemical staining was examined by fluorescent microscopy (BZ9000; Keyence) as previously reported (Yoshikawa et al., 2015). A, Schematic drawing of the SCN. The blue lines indicate the plane of 100-μm sagittal slices that were made. Numbers (–2 ∼ +2) indicate position of the sagittal slices with respect to the midline. B, Images of bioluminescence, immunohistochemical staining for AVP, VIP, and overlay of AVP and VIP. C, Chart comparing robustness of oscillation and expression of AVP and VIP in each slice. Slice IDs indicate good (black) and poor (grey) rhythms. Number of immunopositive cell bodies for VIP and AVP are expressed in symbols. ++, high; +, medium; ±, low; –, none. Robust oscillation requires both AVP and VIP expression. Slices bearing a large number of AVP neurons but lacking VIP showed poor rhythm. The one slice that had both peptides could not classified with respect to rhythmicity due to technical equipment problems. Anatomical analysis of peptide expression was conducted independently and prior to classification of rhythmicity. The number of positive cell bodies were scored as follows. For VIP: >10 cells = ++, 5–9 cells = +, 1–4 cells = ±, no cells = –. For AVP: >20 cells = ++, 5–20 cells = +, 1–5 cells = ±, no cell = –. Download Figure 2-1, TIF file. [file enu-eN-NWR-0078-21-s04.tif]

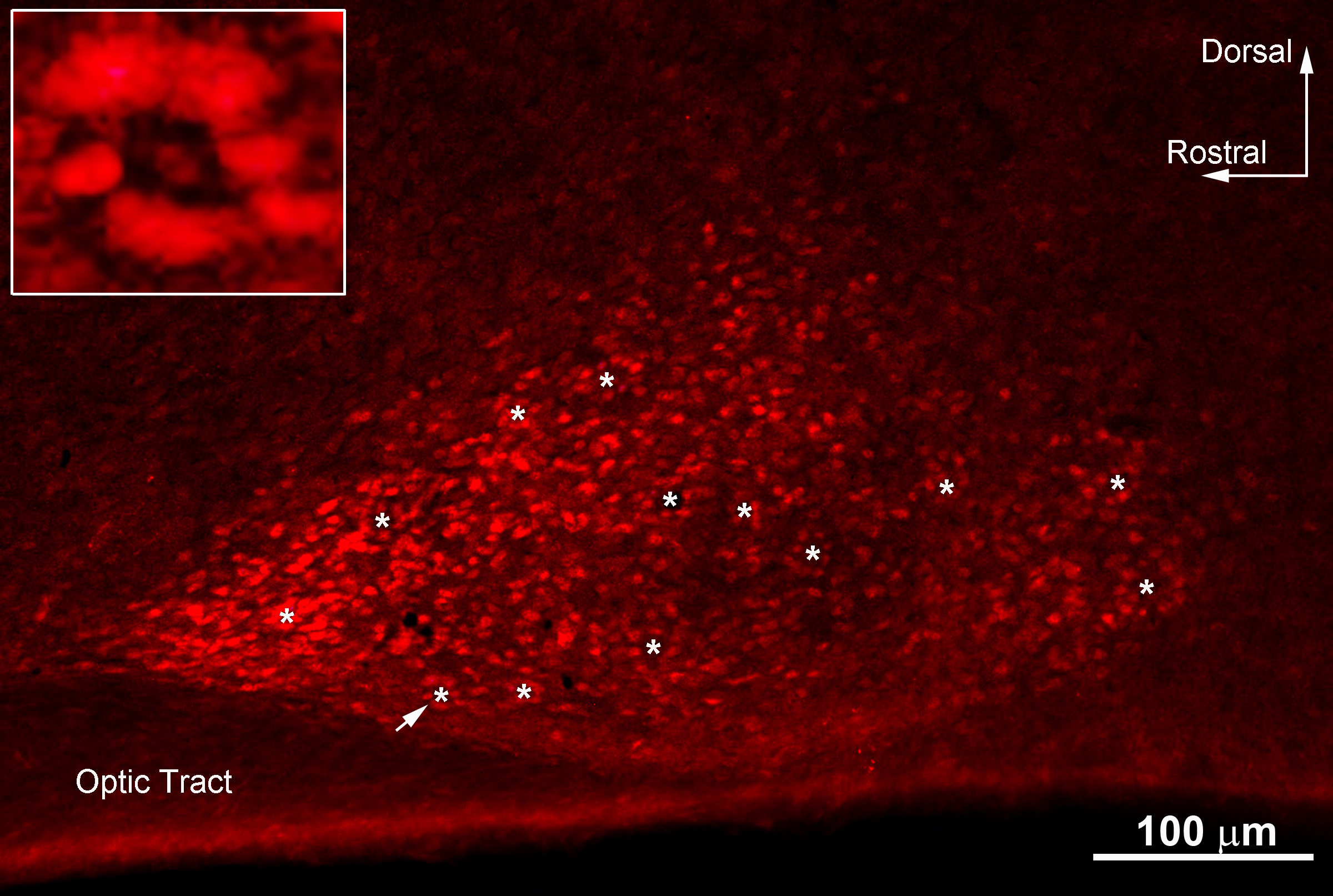

Supplement: Extended data Figure 2-2 — Phaseomes in fixed SCN tissue: photomicrograph of a 50-μm sagittal SCN section immunostained for PER2 (red) at ZT20. White asterisks show the location of phaseomes. The inset is a magnification of the phaseome indicated by the white arrow. Slices were processed for immunocytochemistry as in Riddle et al. (2017). Download Figure 2-2, TIF file. [file enu-eN-NWR-0078-21-s05.tif]

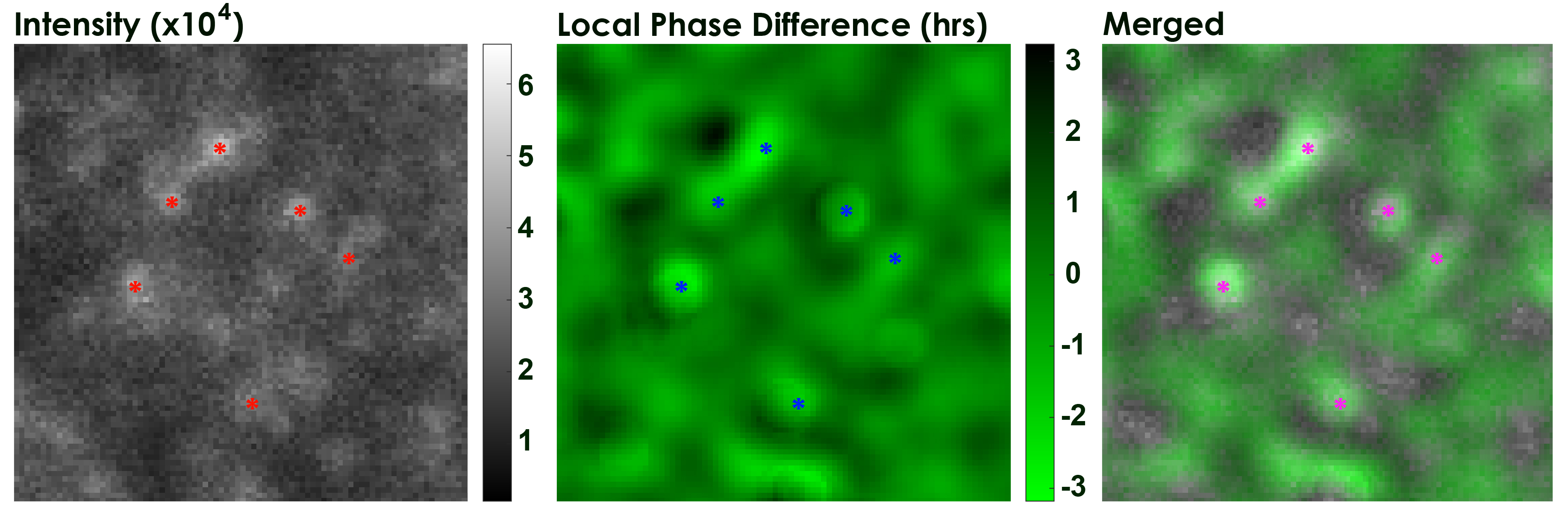

Supplement: Extended data Figure 3-1 — This figure gives a comparison between manual identification of neurons that are possibly out of phase with their surroundings and the results of the local phase difference computations. The left panel shows a region of the SCN in a movie frame near the trough of the mean oscillation of the tissue. In observing the movie, we could identify several neurons (marked with a red asterisk) that seemed to be out of phase with much of the surrounding tissue. In the middle panel, different intensities of green indicate the results of the local phase computations, with the more negative phase differences shown in brighter green. The locations of the neurons from the first panel are marked with blue asterisks where the color scale indicates that they are oscillating between 2 and 3 h behind the surrounding annular region. The right panel shows a merging of the two other panels, demonstrating that the higher intensity areas from the first panel coincides with more negative local phase differences from the second panel. The overlay creates magenta asterisks showing that the placement of the images coincides. Download Figure 3-1, TIF file. [file enu-eN-NWR-0078-21-s06.tif]
